# Altered Pulmonary Lymphatic Development in Infants with Chronic Lung Disease

**DOI:** 10.1155/2014/109891

**Published:** 2014-01-02

**Authors:** Emily M. McNellis, Sherry M. Mabry, Eugenio Taboada, Ikechukwu I. Ekekezie

**Affiliations:** ^1^Division of Neonatal-Perinatal Medicine, The Children's Mercy Hospitals and Clinics, 2401 Gillham Road, Kansas City, MO 64108, USA; ^2^Division of Pathology, The Children's Mercy Hospitals and Clinics, 2401 Gillham Road, Kansas City, MO 64108, USA

## Abstract

Pulmonary lymphatic development in chronic lung disease (CLD) has not been investigated, and anatomy of lymphatics in human infant lungs is not well defined. *Hypothesis*. Pulmonary lymphatic hypoplasia is present in CLD. *Method*. Autopsy lung tissues of eighteen subjects gestational ages 22 to 40 weeks with and without history of respiratory morbidity were stained with monoclonal antipodoplanin and reviewed under light microscopy. Percentage of parenchyma podoplanin stained at the acinar level was determined using computerized image analysis; 9 CLD and 4 control subjects gestational ages 27 to 36 weeks were suitable for the analysis. *Results*. Distinct, lymphatic-specific staining with respect to other vascular structures was appreciated in all gestations. Infants with and without respiratory morbidity had comparable lymphatic distribution which extended to the alveolar ductal level. Podoplanin staining per parenchyma was increased and statistically significant in the CLD group versus controls at the alveolar ductal level (0.06% ± 0.02% versus 0.04% ± 0.01%, 95% CI −0.04% to −0.002%, P < 0.03). *Conclusion*. Contrary to our hypothesis, the findings show that there is an increase in alveolar lymphatics in CLD. It is suggested that the findings, by expanding current knowledge of CLD pathology, may offer insight into the development of more effective therapies to tackle CLD.

## 1. Introduction

Chronic lung disease (CLD), a result of combined oxidative and ventilator induced lung injury, is a major cause of respiratory morbidity in very low birthweight (VLBW) infants [1]. The major pathological findings of blood microvascular hypoplasia and arrested alveolarization [2–6] translate clinically into varying degrees of respiratory insufficiency and impaired pulmonary tissue fluid homeostasis [1, 7]. The latter has been attributed to capillary leak from injured vasculature [8–10] and, in the case of established CLD, increased capillary perfusion pressure from decreased vascular load and/or increased arteriolar muscularization [11].

It is known that maintenance of tissue perfusion homeostasis and response to interstitial fluid burdens in pathological states are dependent on an intact lymphatic system. With regard to the neonatal lung at risk for CLD, investigators have reported increased lymphatic congestion in autopsy lungs from human infants dying of respiratory distress syndrome (RDS) [12] and increased lymphatic flow in sheep models of early CLD [13]. While these studies describe increased reliance on the lymphatic system in the face of evolving CLD, lymphatic microvascular development as it relates to CLD is not known. Moreover, knowledge of anatomy of lymphatics in normal human lungs is limited, especially as there are no data from infant lungs based on immunohistochemical detection of lymphatics [14, 15]. A failure of adequate adaptation and/or development of the pulmonary lymphatic microvasculature may contribute to the pathomorbidity and mortality seen in established CLD.

Pulmonary blood microvascular development is linked with alveolar development and both are impaired in CLD [4, 5]. Since it is likely that blood and lymphatic vascular development in the lungs occur in parallel [16], we *hypothesized* that lymphatic hypoplasia would be present in lungs of infants with CLD when compared to that of age-matched controls. The objectives of this study were to examine pulmonary lymphatic distribution in developing human lungs from normal and diseased infant lung tissue, and, importantly, to quantify and compare the abundance of lymphatic microvasculature at the acinar level in infants with CLD versus controls.

## 2. Materials and Methods

### 2.1. Subjects

A bank of formalin-fixed paraffin-embedded human infant lung autopsy specimens gestational ages 22 to 40 weeks was accessed. On initial selection, subjects were excluded if there was history of congenital anomalies known to affect the pulmonary system, prolonged rupture of membranes >3 days, severe bronchopneumonia, extensive pulmonary hemorrhage, and extreme intrauterine growth restriction. A sample size of eighteen infants was chosen (Table 1). It consisted of 9 infants who died from nonrespiratory causes within 48 to 72 hours of birth with only brief exposure to oxygen and/or ventilation and 9 infants of similar gestational age or postmenstrual age (PMA) at risk or with evidence of moderate to severe chronic lung disease (CLD) at the time of death. From this group, 13 infants with gestational or PMA of 27 to 36 weeks were chosen for quantitative study of acinar lymphatics. The control group consisted of four infants who died from nonrespiratory causes after 1.7 ± 1.3 days of birth at 31 ± 4-week gestational age with a low respiratory severity score [17]. The chronic lung disease (CLD) group consisted of nine infants with evidence of moderate (1 subject) to severe (8 subjects) CLD or at risk for CLD at the time of death after 30 ± 16 days at 30 ± 2-week PMA. CLD was defined as supplemental oxygen requirement at 28 days and those at risk identified as having an elevated respiratory score determined by multiplying the average daily FiO_2_ by the average daily mean airway pressure (MAP) in cm H_2_O and integrating the area under the curve, using the trapezoidal rule, for the total number of days lived [17].

This project was reviewed by the University of Missouri-Kansas City Children's Mercy Pediatric Institutional Review Board and received an exempt status.

### 2.2. Lung Preparation

Lungs were collected from 1988 to 2002. The time from death to autopsy was 15 ± 11 hours. Lungs from five of the subjects used in a previous study in our lab were infused with a heated barium-gelatin mixture at about 70 mm Hg pressure via the pulmonary artery until uniform surface filling of the lungs was appreciated and the pulmonary artery ligated at the infusion pressure. Lungs from the remaining 13 subjects were not barium infused. Both barium and nonbarium filled left lungs were deflated by vacuum, warmed to 38°C, and inflated via the trachea for 72 hours at 24 cm H_2_O pressure with 10% formalin. Sections of the left lung were cut horizontally, 2-3 mm thick, from top to bottom of the lung. Ten to fifteen blocks were chosen at random from the slabs and paraffin embedded.

### 2.3. Immunohistochemical Methods

Three to five 5-*μ*m thick sections of formalin-fixed, paraffin-embedded tissues were mounted on positively charged glass slides and placed in 60°C oven overnight (12 to 18 hours) then subjected to xylene and graded concentrations of ethanol for dewaxing. Slides from all subjects underwent immune staining with monoclonal mouse anti-human podoplanin (Acris Antibodies, Hiddenhausen, Germany) using a modified avidin-biotin-peroxidase method [18]. Podoplanin was chosen for this study, as it appeared to have superior lymphatic specificity in lung tissue when compared to VEGFR-3, LYVE-1, and Prox-1 in a pilot study performed by our lab. 1 : 800, 1 : 400, and 1 : 200 dilutions of podoplanin antibody were tested first to determine optimal concentration to avoid over or understaining of which a 1 : 200 dilution was chosen. Slides were first deparaffinized with xylene twice for 3–5 minutes per pass, placed in 100% alcohol then 95% alcohol for 1–3 minutes per pass, and then rehydrated in deionized H_2_O. Antigen retrieval was enhanced by steaming slides bathed in target retrieval solution (DAKO, Carpinteria, CA) for 20 minutes. After incubation with blocking serum (DAKO, Carpinteria, CA) for 10 minutes, slides were incubated with primary antibody for 1 hour. Endogenous peroxidase was quenched with hydrogen peroxide and slides were then incubated for 30 minutes with biotinylated IgG and avidin-biotin peroxidase complex (Vectastain ABC Elite Kit, Vector Laboratories, Burlingame, CA). Antigenic sites were visualized by the addition of the chromogen 3,3′ diaminobenzidine (DAB), keeping the time in which slides were exposed equal. The slides were then counterstained with Harris hematoxylin (Fisher HealthCare, Houston, TX). Negative control slides were stained using the same procedures omitting the primary antibody.

To confirm podoplanin specificity for lymphatic endothelial cells, slides from all subjects were also immunostained sequentially with a mouse anti-human podoplanin monoclonal antibody (Acris Antibodies GmBH, Herford, Germany) and a mouse anti-human CD31 monoclonal antibody (AbD Serotec, Raleigh, NC) using a polymerized reporter enzyme staining system (Vector Laboratories, Inc., Burlingame, CA). After xylene deparaffinization (2 × 3 minutes) and rehydration in graded alcohols to deionized water, antigen retrieval was performed by steaming slides bathed in target retrieval solution, pH 6.1 (DAKO corporation, Carpinteria, CA) 20 min then cooling 15 minutes at room temperature. Slides were rinsed in deionized water, then were incubated in 3% hydrogen peroxide to block endogenous peroxidase activity, and were blocked with 2.5% normal horse serum for 20 minutes. Sections were incubated with podoplanin antiserum (1 : 200) for 1 hour at room temperature, rinsed in phosphate-buffered saline, pH 7.4 (PBS), and incubated with a micropolymer of active peroxidase coupled to an affinity purified anti-mouse IgG (H + L) secondary antibody for 30 minutes at room temperature. After rinsing with PBS, antigenic sites were visualized by the addition of the chromogen (DAB). Slides were rinsed in tap water and again blocked with 2.5% normal horse serum for 20 minutes. The sections were then incubated with CD31 antiserum (1 : 50) overnight at 4°C. After rinsing with PBS, they were incubated with the same anti-mouse IgG reagent used previously for 30 minutes at room temperature. They were rinsed again with PBS and CD31 antigenic sites were visualized with ImmPACT VIP Substrate (Vector Laboratories, Inc.). Slides were rinsed with tap water and counterstained with methyl green (Vector Laboratories, Inc.).

### 2.4. Image Analysis

Slides stained with podoplanin alone were viewed under standard light microscopy at 4, 10, 20, and 40x magnification and assessed for quality of staining and histology. Specimens with mild to moderate evidence of lung injury were analyzed but those with extensive pulmonary hemorrhage, bronchopulmonary pneumonia, or poorly expanded or overdistended architecture were excluded. Slides from the thirteen subjects chosen for quantitative study were then viewed via light microscopy by an observer blinded to the gestation and clinical status of the subjects at 40x magnification. Lymphatic vessels associated with arterioles at the respiratory bronchiolar and alveolar ductal levels were identified in well-expanded regions in 40 to 60 consecutive fields per slide. Fields with conducting airways or large blood vessels filling more than 50% of the field were skipped. Lymphatic vessels in CLD samples were studied in regions exhibiting typical histological features of CLD including cystic areas and/or regions with thickened interstitium. Using standard image analysis software (analySIS, Soft Imaging System Corp., Lakewood, CO), images were photographed and converted to grey scale and parenchymal tissues with and without podoplanin staining were assigned different thresholds. As podoplanin had selectivity for type I alveolar epithelial cells in some specimens, any stained tissues other than lymphatic vessels were kept out of the field of interest for analysis. The percentage of parenchyma that was podoplanin stained was then measured. Parenchyma in this study included tertiary arterioles, saccules, alveolar ducts, alveolar walls, septae, and air. Measurements were performed on lymphatic vessels that appeared to be in cross-section and associated with an arteriole for better standardization. An analysis was not performed if vessels appeared to be in a significantly oblique or longitudinal orientation; there was too much background staining, artifact, or other positive staining structures in close proximity that could not be excluded from the area of interest. This approach was used to avoid overestimation of actual lymphatic tissue.

### 2.5. Statistical Analysis

Statistical analysis was performed using SPSS version 9.2 (SPSS, Inc.). The average percentage parenchyma and interstitial staining for each specimen was calculated. The average mean and 95% CI for difference in means were then determined between infants with CLD and controls. A *P* value <0.05 was considered significant.

## 3. Results

### 3.1. Pulmonary Lymphatic Distribution in Infants 22- to 40-Week Gestation

**Figure 1:**
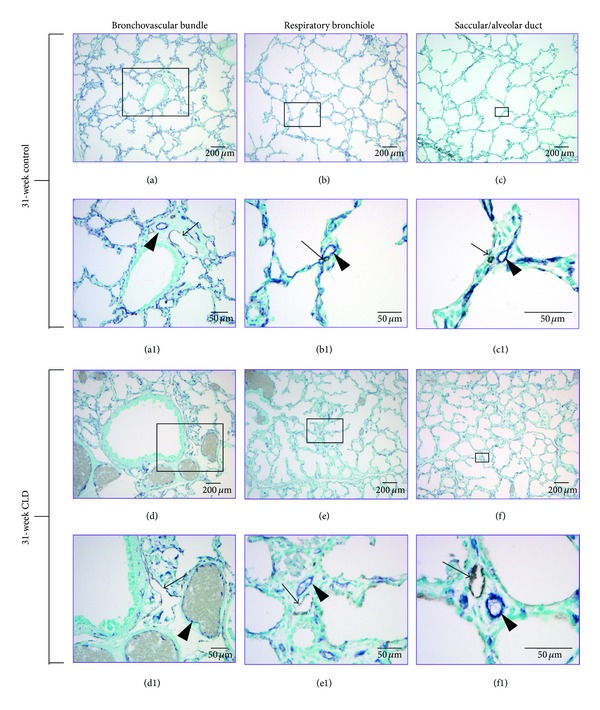
Lung specimens from a 31-week control infant ((a)–(c1)) and 31-week infant with moderate CLD ((d)–(f1)). Bronchovascular bundle ((a), (d) and (a1), (d1)), respiratory bronchiolar ((b), (e) and (b1), (e1)), and alveolar ductal ((c), (f) and (c1), (f1)) levels are displayed. ((a1), (b1), (c1)) and ((d1), (e1),(f1)) are higher magnifications (10x, 20x, 40x, resp.) of regions outlined in ((a), (b), (c)) and ((d), (e), (f)), respectively (magnification 4x). Slides are double immunostained with anti-human podoplanin and anti-human CD31 antibodies. Podoplanin and CD31 stains are selective for lymphatic and blood vasculature, respectively. Lymphatic vessels are stained brown (arrows) and are associated with arteries and arterioles. Arteries and arterioles are stained purple (arrowheads).

**Figure 2:**
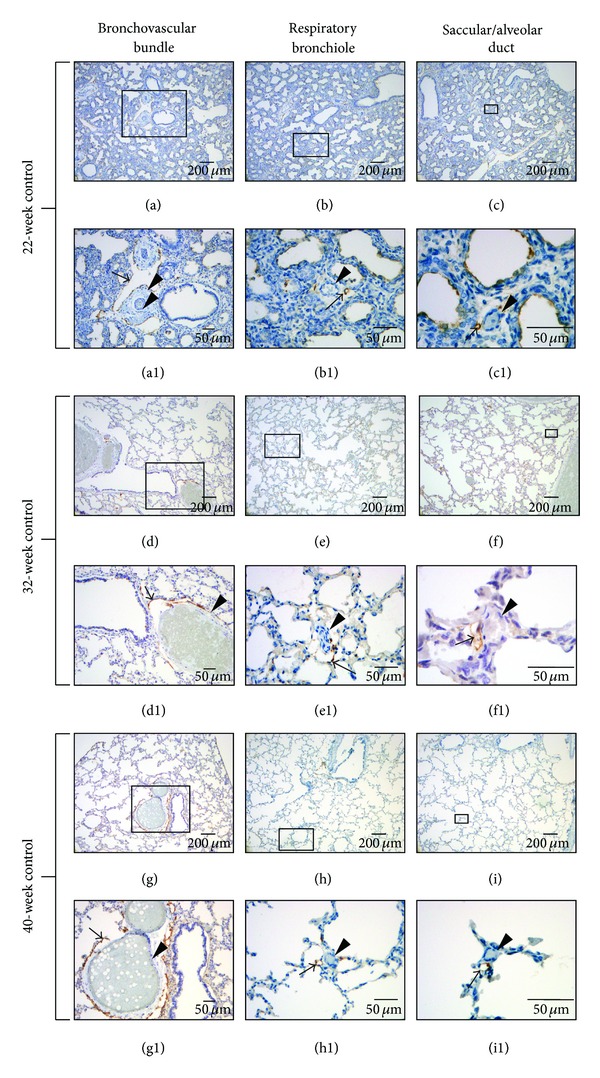
Pulmonary lymphatic distribution at the bronchovascular bundle ((a), (d), (g) and (a1), (d1), (g1)), respiratory bronchiolar ((b), (e), (h) and (b1), (e1), (h1)), and saccular/alveolar ductal ((c), (f), (i) and (c1), (f1), (i1)) levels in 22- ((a)–(c1)), 32- ((d)–(f1)), and 40- ((g)–(i1)) week gestation infants without a history of respiratory morbidity. ((a1), (b1), (c1)), ((d1), (e1) (f1)) and ((g1), (h1), (i1)) are higher magnifications (10x, 20x, 40x, respectively) of regions outlined in ((a), (b), (c)), ((d), (e), (f)), and ((g), (h), (i)), respectively (magnification 4x). Lymphatic vessels are stained with monoclonal mouse anti-human podoplanin antibody (arrows) and are associated with red blood cell or barium-filled arteries and arterioles (arrowheads). Lymphatics are well-developed at the bronchovascular and respiratory bronchiolar levels at all gestations with a paucity of staining in the distal parenchyma in the 22-week subject. Additional selectivity is seen with saccular epithelial staining in the 22-week subject ((b1), (c1)) that is absent in older gestational ages. Lymphatic staining is not appreciated beyond the alveolar ductal level in any gestation.

Immunostaining of podoplanin in fetal and infant lung tissue displayed distinct lymphatic-specific staining with respect to other vascular structures regardless of gestational age or presence of lung injury. Specificity was confirmed with double immunostaining (Figure 1) with mouse anti-human podoplanin and mouse anti-human CD31 antibody which displayed lymphatic-specific staining with podoplanin while other vascular structures (arteries, arterioles, capillaries, and veins) stained with CD31 only. In connective tissue sheaths supporting bronchovascular bundles (Figures 2(a), (a1), (d), (d1), (g), and (g1)) along with interlobular and pleural regions, lymphatic vessels are extremely dense, usually associated with blood vasculature and most have open lumens. In bronchovascular bundle sheaths, lymphatic vessels have extremely thin, irregular serpentine walls investing conducting airways and blood vasculature extensively (Figures 2(a), (a1), (d), (d1), (g), and (g1)). In interlobular planes, lymphatics investing venous structures are appreciated as well as single lymphatic vessels that traverse great lengths with occasional connections with lymphatic vessels from other regions. Lymphatic communication between bronchovascular bundles and interlobular planes can be appreciated. In addition to lymphatic vessels that are associated with veins, pleural tissue also possesses large, variably shaped lymphatics not associated with blood vasculature that occasionally connect with interlobular lymphatics. Lymphatic vascularization in all of these regions appears to be well developed even in the lowest gestational ages studied of 22 to 25 weeks.

**Figure 3:**
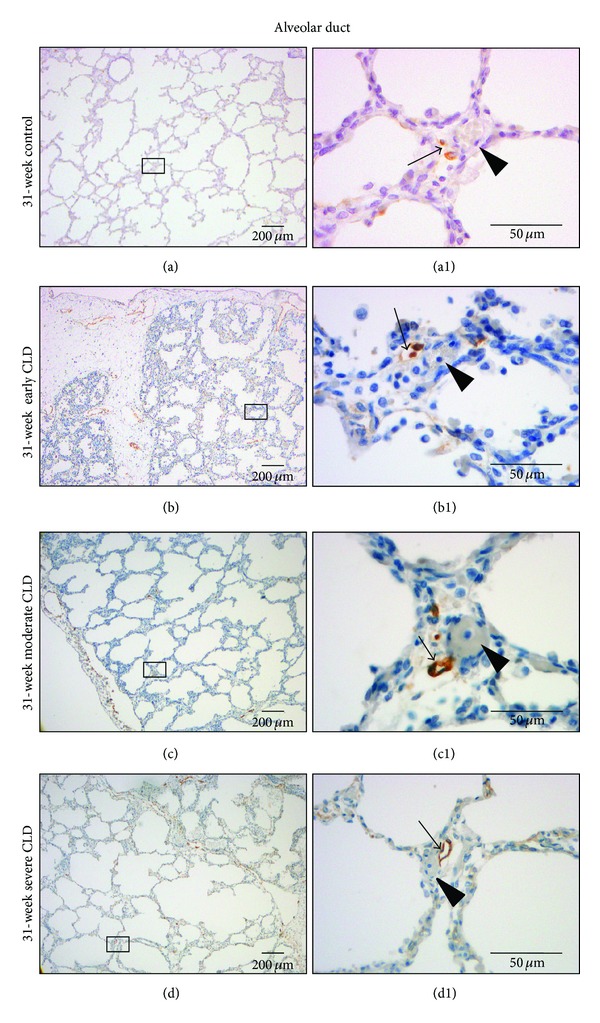
Lymphatic vascular staining with monoclonal antihuman podoplanin antibody at the alveolar ductal level in a 31-week infant without history of respiratory morbidity ((a), (a1)) and 3 infants with a postmenstrual age of 31 weeks with early CLD ((b), (b1)), moderate CLD ((c), (c1)), and severe CLD ((d), (d1)). ((a1), (b1), (c1), (d1)) are higher magnifications (40x) of regions outlined in ((a), (b), (c), (d)), respectively (magnification 4x). Note presence of interstitial thickening and presence of inflammatory cells in ((b), (b1)), decreased alveolarization ((c), (c1)), and cystic distortion ((d), (d1)) as CLD evolves. Lymphatic vessels (arrows) are associated with red blood cell or barium-filled arterioles (arrowheads).

Beyond connective tissue planes, lymphatic vessels are consistently associated with arterioles and extend peripherally from bronchovascular bundles as far as the saccular/alveolar ductal level (Figures 2(c), (c1), (f), (f1), (i), and (i1)). Lymphatic microvasculature at the alveolar ductal level is present in infants with and without a history of respiratory morbidity (Figure 3). Lymphatics become less extensive and their presence more variable moving peripherally from the terminal bronchiole to the alveolar ductal level. Lymphatic vessels at this level usually appear to have closed lumens with occasional communication with interlobular lymphatics. These connections are more apparent past 24- to 25-week gestation. More abundant lymphatic staining in peripheral regions of the lung is noted in advanced gestational ages (>30 weeks). No distinct interstitial staining is present within the alveolar septa with antipodoplanin antibodies at any gestational age or under different pathological conditions.

In addition to lymphatic vasculature, antipodoplanin antibody was found to be selective for bronchial-associated chondrocytes (not shown), mesothelium, and saccular/type I alveolar epithelial cells (Figures 2(b1) and (c1)). Interestingly, staining of the distal airway epithelium again became apparent in infants with gestations greater than 32 weeks with a history of respiratory morbidity. With the exception of the latter, staining of bronchial and mesothelial structures was independent of gestation or lung injury. Bronchial epithelial basement membrane occasionally stained in lower gestations (not shown).

### 3.2. Quantification of Pulmonary Lymphatics at the Acinar Level in Infants with CLD Compared to Age-Matched Controls

**Figure 4:**
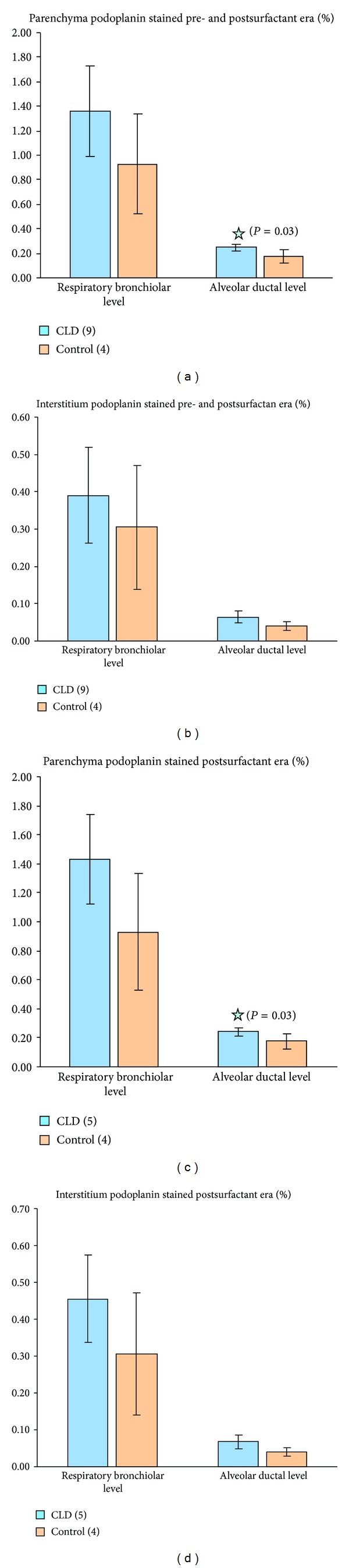
Percent parenchyma and interstitium podoplanin stained (representing lymphatic tissue) at the respiratory bronchiolar and alveolar ductal levels in infants with CLD versus controls. (a) and (b) include CLD infants from both pre- and postsurfactant eras; (c) and (d) include infants from postsurfactant era only. Podoplanin staining is consistently increased at both levels in infants with CLD reaching statistical significance at the alveolar ductal level with percent parenchyma stained ((a) and (c)).

Average parenchyma podoplanin stained at the saccular/alveolar ductal level was increased and statistically significant in the CLD group compared to controls (0.06% ± 0.02% versus 0.04% ± 0.01%, 95% CI −0.04% to −0.002%, *P* = 0.03) (Figure 4). Staining at the respiratory bronchiolar level was also increased but not statistically significant in the CLD group versus controls (0.25% ± 0.03% versus 0.18% ± 0.05% CI −0.15 to 0.01, *P* = 0.06). Because interstitial thickening is seen in CLD and parenchymal measurements included air, the percent interstitium podoplanin stained was also calculated to see if podoplanin staining remained relatively increased in the CLD group. Average interstitium podoplanin stained remained increased at the respiratory bronchiolar and saccular/alveolar ductal regions in the CLD group versus controls but did not reach statistical significance (1.36% ± 0.37% versus 0.93% ± 0.4% CI −1.03 to 0.16, *P* = 0.12 for the respiratory bronchiolar level and 0.39% ± 0.13% versus 0.31% ± 0.17% CI −0.34 to 0.16, *P* = 0.4 for the saccular/alveolar ductal level).

Four of the nine infants with CLD did not receive surfactant (specimens were collected prior to 1990) and arguably may have worse histological abnormalities than those that did receive surfactant. An additional analysis was thus performed comparing only those infants with CLD that had received surfactant and control infants. This analysis continued to show increased parenchymal and interstitial podoplanin staining in the CLD group versus controls at the respiratory bronchiolar and saccular/alveolar ductal levels reaching significance at the alveolar ductal level with percent parenchyma stained (0.07% ± 0.02% versus 0.04% ± 0.01 CI −0.05 to −0.003, *P* = 0.03). Percent parenchyma stained at respiratory bronchiolar level in CLD group versus controls was 0.24% ± 0.3% versus 0.18% ± 0.05% CI −0.14 to 0.01, *P* = 0.07. The percent interstitium podoplanin stained in the postsurfactant CLD group versus controls was again increased in infants with CLD but did not reach significance (1.43% ± 0.31% versus 0.93% ± 0.4% CI −1.11 to 0.11, *P* = 0.09 at the respiratory bronchiolar level and 0.46% ± 0.12% versus 0.31% ± 0.17% CI −0.4 to 0.1, *P* = 0.2 at the alveolar ductal level).

## 4. Discussion

To our knowledge, this is the first study describing pulmonary lymphatic distribution in human infant lungs using podoplanin as surrogate marker to identify lymphatics. Moreover, we examined pulmonary lymphatic development in the setting of chronic lung disease (CLD) by quantifying podoplanin staining at the acinar level in subjects with and without CLD. In both pre- and postsurfactant era groups of CLD infants, podoplanin staining at the acinar level was increased when compared to age-matched controls. As lymphatic development in the face of CLD has not been previously reported, this finding adds to the current understanding of the pathology in CLD and thus may provide additional insight into the development of effective therapies for the condition.

Qualitative comparison of the infants with and without a history of respiratory morbidity revealed comparable temporospatial distribution of the pulmonary lymphatics into pleural, interlobular, bronchovascular, and acinar vessels at gestational ages 22 to 40 weeks. Those lymphatic networks within the pleura, interlobular septae, and connective tissue sheaths supporting bronchovascular bundles appeared well developed even in the lowest gestational age of 22 weeks. This degree of development is not surprising considering that the bronchopulmonary segments are complete by 16 to 18 weeks and there is evidence that airway, vascular, and lymphatic development occur in a coordinated fashion [16, 19]. Consistent with reports from adult lung studies [14, 15, 20–24], we find lymphatic vessels extended no further than the alveolar ductal level with the air blood barrier devoid of lymphatics regardless of age or presence of injury.

Quantitative study of lymphatic vascularization in infants with CLD versus age-matched controls was directed at the acinar level where oxidative and positive pressure-induced injury has the most deleterious effect in preterm lungs. The increased podoplanin staining that we observed at this level in CLD infants compared to controls suggests that increased lymphatics are present. Mechanisms driving this may include either neoproliferation or recruitment of existing lymphatics.

Lymphangiogenesis, or the growth of new lymphatic vessels, is orchestrated by Prox-1, a homeobox transcription factor, and vascular endothelial growth factors (VEGF) C and D [25–27]. Pulmonary lymphangiogenesis outside normal development has been observed as a response to chronic inflammatory processes associated with transplantation, wound healing, and tumor growth/metastasis [28, 29]. Indeed, it was recently reported that COPD patients have increased number of alveolar lymphatics [30, 31]. Increased lymphangiogenesis in the setting of CLD may follow a similar pathway as key inflammatory mediators such as NF-kappaB [32] and elevated numbers of VEGF-C producing alveolar macrophages have been demonstrated in tracheal aspirate fluid from premature infants that later develop CLD [33].

In addition to proliferation, the apparent increase in lymphatics observed in infants with CLD may alternatively be due to the recruitment of existing lymphatic vessels. Functional impairment of fluid homeostasis may be related to the loss of normal architecture and elastic properties of the lung upon which lymphatics rely for effective uptake and removal of fluids. In this case, the capacity of an otherwise normal lymphatic system may be exceeded [22]. Illustrating recruitment of lymphatic reserves in the event of acute or chronic lung injury, scanning electron microscopy (SEM) studies of rat lungs following ventilator-induced injury have displayed identical lymphatic structures in both acute and chronic states of pulmonary edema [34]. In addition, lymphatic vessels in this and other studies have been identified by airway or vascular perfusion with plastic resinous compounds that do not reliably dilate microlymphatics in normal as opposed to the inflamed lung [34, 35]. These observations suggest that only a fraction of the existing lymphatic system's capacity is utilized for routine maintenance of pulmonary fluid homeostasis. If podoplanin expression is linked to the degree of lymphatic function, increased podoplanin staining in infants with CLD could simply signify the number of lymphatic vessels in use.

Despite podoplanin's lymphatic specificity relative to other vascular structures in our study lungs, saccular/type I epithelial cells were also found to stain positively with specific temporal and pathological patterns. T1-alpha, a podoplanin homolog estimated to be 50% identical at the nucleotide level [36], is present exclusively in type I epithelial cells in mice at all levels of development. In our study of human lung, however, staining of saccular/type I alveolar cells was seen consistently in gestations ≤24 weeks or in the presence of severe lung injury with otherwise sparse to no staining after 32-week gestation.

From a developmental standpoint, podoplanin may contribute to the transition and maturation of cuboidal epithelial cells to a more flattened type I phenotype [37–40] and/or contribute to secondary septation in those infants ≤24 weeks. With regard to lung injury, hyperoxia exposure in the rat lung has been associated with an increased expression of the podoplanin homolog T1-alpha [41], and in the mouse lung, increased T1-alpha staining of Type I alveolar cells [42]. If conserved expression of podoplanin exists in human saccular or Type I alveolar cells with exposure to hyperoxia, the staining patterns in this study support upregulation of podoplanin in the presence of lung injury specifically in RDS and CLD.

Although pulmonary vascular hypoplasia is predominately thought to be a major finding in CLD [2–6] other studies suggest that while microvasculature remains dysmorphic, remodeling of the remaining septae may establish a normal capillary load [43, 44] and eventually pulmonary function in infants with CLD. While our study compliments this notion from a lymphatic standpoint, other structural differences in CLD may have influenced our findings. As many of the distal acinar structures have been obliterated or developmentally interrupted in CLD, it is the more proximal structures that are left behind to adapt. In our study, it is possible that the more distal acinar structures in control subjects have been compared to the adapted proximal structures in the CLD subjects potentially making the amount of acinar lymphatic tissue in CLD appear larger. This raises the question as to whether an adaptation has really taken place (by way of proliferation) and may still imply a relative paucity of acinar level lymphatics when considering the lung parenchyma as a whole.

Several other limitations of this study exist which first include the small sample sizes for both control and CLD groups. Obtaining optimally preserved, nonedematous human autopsy specimens is challenging at best making even the control subjects suboptimal representatives of “normal” lung tissue. In addition, a more appropriate control group for the subjects with severe CLD used in our study would be those infants with mild or moderate CLD born at similar gestational ages. There is naturally a paucity of midgestation infants falling in this category however due to improved survival. This study also only represents those infants with CLD that died which makes the finding of increased lymphatics difficult to extrapolate to those that survived.

Podoplanin staining of other lung structures in this study poses a limitation to accurate measurement of lymphatics. Although other stained structures were kept out of the field of interest, this may have affected the relative amount of interstitium included in the measurement especially for the CLD infants as they tended to have increased Type 1 alveolar cell staining. This approach potentially overestimates the amount of podoplanin staining per interstitium.

A larger number of subjects studied under rigorous stereologic techniques will be needed to overcome many of these limitations.

## 5. Conclusion

This study shows that chronic lung disease is associated with increased lung lymphatics at the alveolar ductal level. As lung lymphatic vascularization in the face of CLD has not been previously reported, it is suggested that this observation not only adds to the current understanding of the pathology in CLD but may also open insight into new therapeutic approaches to tackle the condition.

## Figures and Tables

**Table 1:** Clinical variables of controls and infants with CLD.

	Controls		CLD*
	Qualitative	Quantitative	
Number	9	4	9
Gestational age, weeks	31 ± 6^a^	31 ± 4	26 ± 2
Postmenstrual age (PMA)	NA	NA	30 ± 2
Birth weight, kg	1.926 ± 1.2	1.474 ± 0.894	0.819 ± 0.222
Ventilator days	1.6 ± 1.0	1.7 ± 1.3	29.6 ± 19
Days lived	1.6 ± 1.0	1.7 ± 1.3	30 ± 16
% antenatal steroids	11	0	42
% surfactant	33	50	71
% RDS (moderate to severe)	0	0	71
% postnatal steroids	11	0	71
Cause of death			
Respiratory failure	0	0	3
Extreme immaturity (died in DR)	1	0	0
Intracranial hemorrhage, grade IV	1	0	0
NEC	0	0	2
DIC/other hemorrhage	2	1	1
HIE/encephalomalacia	3	2	0
Sepsis/pneumonia	1	1	1
Other	1	0	2

^a^Average ± standard deviation.

*Complete clinical data available for 7 CLD infants.

## References

[1] American Thoracic Society Documents (2003). Statement on the care of the child with chronic lung disease of infancy and childhood. *American Journal of Respiratory and Critical Care*.

[2] Lassus P, Ristimäki A, Ylikorkala O, Viinikka L, Andersson S (1999). Vascular endothelial growth factor in human preterm lung. *American Journal of Respiratory and Critical Care Medicine*.

[3] Lassus P, Turanlahti M, Heikkilä P (2001). Pulmonary vascular endothelial growth factor and Flt-1 in fetuses, in acute and chronic lung disease, and in persistent pulmonary hypertension of the newborn. *American Journal of Respiratory and Critical Care Medicine*.

[4] Jakkula M, Le Cras TD, Gebb S (2000). Inhibition of angiogenesis decreases alveolarization in the developing rat lung. *American Journal of Physiology: Lung Cellular and Molecular Physiology*.

[5] Maniscalco WM, Watkins RH, Pryhuber GS, Bhatt A, Shea C, Huyck H (2002). Angiogenic factors and alveolar vasculature: development and alterations by injury in very premature baboons. *American Journal of Physiology: Lung Cellular and Molecular Physiology*.

[6] Kallapur SG, Bachurski CJ, Le Cras TD, Joshi SN, Ikegami M, Jobe AH (2004). Vascular changes after intra-amniotic endotoxin in preterm lamb lungs. *American Journal of Physiology: Lung Cellular and Molecular Physiology*.

[7] Adams EW, Harrison MC, Counsell SJ (2004). Increased lung water and tissue damage in bronchopulmonary dysplasia. *Journal of Pediatrics*.

[8] Carlton DP, Cummings JJ, Scheerer RG, Poulain FR, Bland RD (1990). Lung overexpansion increases pulmonary microvascular protein permeability in young lambs. *Journal of Applied Physiology*.

[9] Watts CL, Fanaroff AA, Bruce MC (1997). Elevation of fibronectin levels in lung secretions of infants with respiratory distress syndrome and development of bronchopulmonary dysplasia. *Journal of Pediatrics*.

[10] Groneck P, Gotze-Speer B, Oppermann M, Eiffert H, Speer CP (1994). Association of pulmonary inflammation and increased microvascular permeability during the development of bronchopulmonary dysplasia: a sequential analysis of inflammatory mediators in respiratory fluids of high-risk preterm neonates. *Pediatrics*.

[11] Bland RD, Albertine KH, Carlton DP (2000). Chronic lung injury in preterm lambs: abnormalities of the pulmonary circulation and lung fluid balance. *Pediatric Research*.

[12] Lauweryns JM, Claessens S, Boussauw L (1968). The pulmonary lymphatics in neonatal hyaline membrane disease. *Pediatrics*.

[13] Bland RD, Ling CY, Albertine KH (2003). Pulmonary vascular dysfunction in preterm lambs with chronic lung disease. *American Journal of Physiology: Lung Cellular and Molecular Physiology*.

[14] Kambouchner M, Bernaudin J-F (2009). Intralobular pulmonary lymphatic distribution in normal human lung using D2-40 antipodoplanin immunostaining. *Journal of Histochemistry and Cytochemistry*.

[15] Sozio F, Rossi A, Weber E (2012). Morphometric analysis of intralobular, interlobular and pleural lymphatics in normal human lung. *Journal of Anatomy*.

[16] Mallory BP, Mead TJ, Wiginton DAF, Kulkarni RM, Greenberg JM, Akeson AL (2006). Lymphangiogenesis in the developing lung promoted by VEGF-A. *Microvascular Research*.

[17] Thibeault DW, Mabry SM, Ekekezie II, Truog WE (2000). Lung elastic tissue maturation and perturbations during the evolution of chronic lung disease. *Pediatrics*.

[18] Hsu SM, Raine L, Fanger H (1981). Use of Avidin-Biotin-Peroxidase Complex (ABC) in Immunoperoxidase Techniques: a comparison between ABC and unlabeled antibody (PAP) procedures. *Journal of Histochemistry and Cytochemistry*.

[19] van Tuyl M, Liu J, Wang J, Kuliszewski M, Tibboel D, Post M (2005). Role of oxygen and vascular development in epithelial branching morphogenesis of the developing mouse lung. *American Journal of Physiology: Lung Cellular and Molecular Physiology*.

[20] Lauweryns JM, Boussauw L (1969). The ultrastructure of pulmonary lymphatic capillaries of newborn rabbits and of human infants. *Lymphology*.

[21] Peao MND, Aguas AP, de Sa CM, Pereira AS, Grande NR (1993). Scanning electron microscopy of the deep lymphatic network of the murine lung as viewed in corrosion casts. *Lymphology*.

[22] Marchetti C, Poggi P, Clement MG, Aguggini G, Piacentini C, Icaro- Cornaglia A (1994). Lymphatic capillaries of the pig lung: TEM and SEM observations. *Anatomical Record*.

[23] Schraufnagel DE (1992). Forms of lung lymphatics: a scanning electron microscopic study of casts. *Anatomical Record*.

[24] Schraufnagel DE, Basterra JL, Hainis K, Sznajder JI (1994). Lung lymphatics increase after hyperoxic injury: an ultrastructural study of casts. *American Journal of Pathology*.

[25] Mäkinen T, Alitalo K (2002). Molecular mechanisms of lymphangiogenesis. *Cold Spring Harbor Symposia on Quantitative Biology*.

[26] Podgrabinska S, Braun P, Velasco P (2002). Molecular characterization of lymphatic endothelial cells. *Proceedings of the National Academy of Sciences of the United States of America*.

[27] Hirakawa S, Hong Y-K, Harvey N (2003). Identification of vascular lineage-specific genes by transcriptional profiling of isolated blood vascular and lymphatic endothelial cells. *American Journal of Pathology*.

[28] Ji RC (2007). Lymphatic endothelial cells, inflammatory lymphangiogenesis, and prospective players. *Current Medicinal Chemistry*.

[29] Baluk P, Tammela T, Ator E (2005). Pathogenesis of persistent lymphatic vessel hyperplasia in chronic airway inflammation. *Journal of Clinical Investigation*.

[30] Hardavella G, Tzortzaki EG, Siozopoulou V (2012). Lymphangiogenesis in COPD: another link in the pathogenesis of the disease. *Respiratory Medicine*.

[31] Mori M, Andersson CK, Graham GJ, Löfdahl CG, Erjefält JS (2013). Increased number and altered phenotype of lymphatic vessels in peripheral lung compartments of patients with COPD. *Respiratory Research*.

[32] Aghai ZH, Kode A, Saslow JG (2007). Azithromycin suppresses activation of nuclear factor-kappa B and synthesis of pro-inflammatory cytokines in tracheal aspirate cells from premature infants. *Pediatric Research*.

[33] Janér J, Lassus P, Haglund C, Paavonen K, Alitalo K, Andersson S (2006). Pulmonary vascular endothelial growth factor-C in development and lung injury in preterm infants. *American Journal of Respiratory and Critical Care Medicine*.

[34] Schraufnagel DE, Agaram NP, Faruqui A (2003). Pulmonary lymphatics and edema accumulation after brief lung injury. *American Journal of Physiology: Lung Cellular and Molecular Physiology*.

[35] Hainis KD, Sznajder JI, Schraufnagel DE (1994). Lung lymphatics cast from the airspace. *American Journal of Physiology: Lung Cellular and Molecular Physiology*.

[36] Ma T, Yang B, Matthay MA, Verkman AS (1998). Evidence against a role of mouse, rat, and two cloned human T1*α* isoforms as a water channel or a regulator of aquaporin-type water channels. *American Journal of Respiratory Cell and Molecular Biology*.

[37] Schacht V, Ramirez MI, Hong Y-K (2003). T1*α*/podoplanin deficiency disrupts normal lymphatic vasculature formation and causes lymphedema. *EMBO Journal*.

[38] Breiteneder-Geleff S, Matsui K, Soleiman A (1997). Podoplanin, novel 43-kd membrane protein of glomerular epithelial cells, is down-regulated in puromycin nephrosis. *American Journal of Pathology*.

[39] Ramirez MI, Millien G, Hinds A, Cao Y, Seldin DC, Williams MC (2003). T1*α*, a lung type I cell differentiation gene, is required for normal lung cell proliferation and alveolus formation at birth. *Developmental Biology*.

[40] McElroy MC, Kasper M (2004). The use of alveolar epithelial type I cell-selective markers to investigate lung injury and repair. *European Respiratory Journal*.

[41] Cao Y-X, Ramirez MI, Williams MC (2003). Enhanced binding of Sp1/Sp3 transcription factors mediates the hyperoxia-induced increased expression of the lung type I cell gene T1*α*. *Journal of Cellular Biochemistry*.

[42] Yee M, Vitiello PF, Roper JM (2006). Type II epithelial cells are critical target for hyperoxia-mediated impairment of postnatal lung development. *American Journal of Physiology: Lung Cellular and Molecular Physiology*.

[43] Thibeault DW, Mabry SM, Norberg M, Truog WE, Ekekezie II (2004). Lung microvascular adaptation in infants with chronic lung disease. *Biology of the Neonate*.

[44] de Paepe ME, Mao Q, Powell J (2006). Growth of pulmonary microvasculature in ventilated preterm infants. *American Journal of Respiratory and Critical Care Medicine*.

